# Insights on the Bioaccessibility of Natural Pigments from Diatom *Chaetoceros calcitrans*

**DOI:** 10.3390/molecules27103305

**Published:** 2022-05-21

**Authors:** Tatiele C. do Nascimento, Pricila N. Pinheiro, Andrêssa S. Fernandes, Patrícia A. Caetano, Eduardo Jacob-Lopes, Leila Q. Zepka

**Affiliations:** Department of Food Technology and Science, Federal University of Santa Maria (UFSM), Santa Maria 97105-900, RS, Brazil; tatielecasagrande@gmail.com (T.C.d.N.); pricila.nass@gmail.com (P.N.P.); andressa.asfs@gmail.com (A.S.F.); pati.caetano98@gmail.com (P.A.C.); ejacoblopes@gmail.com (E.J.-L.)

**Keywords:** brown microalgae, bioactive compounds, carotenoids, chlorophylls, in vitro digestion, bioaccessibility

## Abstract

This study aimed to investigate the bioaccessibility of carotenoids and chlorophylls from the biomass of microalgae *Chaetoceros calcitrans*. The samples were submitted to an in vitro digestion protocol, and the compounds were determined by HPLC-PDA-MS/MS. A total of 13 compounds were identified in all tests. After in vitro digestion, the relative bioaccessibility of carotenoids and chlorophylls ranged from 4 to 58%. The qualitative profile of carotenoids reflected the initial sample, with all-*E*-zeaxanthin (57.2%) being the most bioaccessible compound, followed by all-*E*-neochrome (31.26%), the latter being reported for the first time in the micellar fraction. On the other hand, among the chlorophylls only pheophytin a (15.01%) was bioaccessible. Furthermore, a chlorophyll derivative (Hydroxypheophytin a’) was formed after in vitro digestion. Considering all compounds, xanthophylls (12.03%) and chlorophylls (12.22%) were significantly (*p* < 0.05) more bioaccessible than carotenes (11.22%). Finally, the considerable individual bioaccessibilities found, especially for zeaxanthin, demonstrate the bioactive potential of this bioresource. However, the large reduction in the totality of compounds after in vitro digestion suggests that additional technological strategies should be explored in the future to increase the efficiency of micellarization and enhance its bioactive effects.

## 1. Introduction

As the world’s population becomes more aware of health and global sustainability issues, the potential for microalgae-based processes and products to contribute to solutions to these issues is becoming evident. These microorganisms have been considered potential bioresources to meet the population’s growing needs for a supply of healthier, natural, and sustainable food products, especially fine chemical compounds with potential health-promoting effects [1,2].

Microalgae are acknowledged as one of the most promising renewable resources on the planet. They have some highly relevant characteristics, such as their rapid growth rate and the ability to survive in adverse conditions and substantially contribute to the capture of atmospheric CO_2_ [3,4]. In addition, the countless species of microalgae already cataloged present a highly diversified biochemical composition that includes a multitude of valuable biomolecules [5]. Among these species, *Chaetoceros calcitrans*, belonging to the Chaetocerotaceae family, is a diatom that contains large amounts of natural antioxidants such as carotenoids and chlorophylls [6,7].

Carotenoids and chlorophylls constitute groups of large and complex compounds ubiquitous in microalgae species [8,9]. Many of these structures have exceptional antioxidant capabilities that are continually associated with important biological and functional properties [10,11]. The primary use of these phytochemicals is as natural pigments with wide application in the most varied industrial sectors [12]. However, due to their bioactive properties, which are constantly being demonstrated, these natural pigments are recognized as compounds with high added value, which intensifies their application in food products for health, functional and nutraceutical purposes [13,14,15].

However, for bioactive compounds to exert some activity at the biological level, these molecules must be bioaccessible for intestinal uptake and subsequent systemic distribution in the human body [16,17]. Thus, the bioaccessibility of carotenoids and chlorophylls is considered an essential area of study which is fundamental to understanding their nutritional and functional values and optimizing their applications.

Bioaccessibility is dependent on the degree of release, solubilization, and incorporation of intracellular compounds in mixed-bile-salt micelles [18]. For microalgae, the step involving intracellular release of the compounds is reported as the main limiting factor for bioaccessibility due to the structural and physicochemical properties that contribute to a more rigid cell wall [19,20]. Process intensification technologies such as ultrasoundthat trigger the partial release of molecules through cell disruption have been suggested as strategies to enhance the bioaccessibility of carotenoids and chlorophylls [21,22].

Considering these aspects of bioaccessibility, the objective of this work was to evaluate the bioaccessibility of carotenoids and chlorophylls of ultrasonicated biomass of *Chaetoceros calcitrans*, following an in vitro digestion protocol.

## 2. Results and Discussion

### 2.1. Pigments Composition before and after Digestion in vitro

A total of 13 compounds were separated in all assays with the microalgae *C. calcitrans* (see Table 1). Identification was based on chemical evidence provided by chromatographic analysis such as elution order and UV-Vis characteristics and was confirmed by MS/MS experiments (The representative chromatograms HPLC-PDA and PDA-MS/MS (MRM) spectra can be found in Supplementary Materials). In addition, pigments were identified or provisionally identified based on a detailed description previously reported for different microalgae species [21,23,24,25,26].

The microalgae cell wall is the first barrier to the effective use of bioactive compounds from this promising group of microorganisms. However, previous studies have demonstrated the efficiency of using ultrasound to increase micellar incorporation of microalgae compounds such as *Chlorella vulgaris*, *Chlamydomonas reinhardtii*, and *Scenedesmus obliquus* [19,21,22]. Therefore, the original content of carotenoids and chlorophylls of the ultrasonicated dried biomass of *C. calcitrans* before digestion (initial content) and the micellar fraction after digestion, are shown in Table 2.

From a quantitative point of view, the initial extract of carotenoids showed seven compounds, totaling 239.88 ± 1.82 µg·g^−1^, of which all-*E*-echinenone (116.65 ± 1.52 µg·g^−1^) was the most abundant, followed by all-*E*-β-carotene (55.44 ± 0.25 µg·g^−1^). On the other hand, the initial chlorophyll extract presented five compounds, making a total of 3944.16 ± 41.56 µg·g^−1^; among these, pheophytin a (3257.52 ± 40.23 µg·g^−1^) was the most abundant.

In general, after digestion simulation, the qualitative profile of carotenoids reflected that of the initial sample. On the other hand, only one of the chlorophylls in the initial extract was identified after digestion. Among the chlorophylls, the derivated compound identified as hydroxypheophytin a’ was detected only in the micellar fraction.

Possibly, this chlorophyll derivative was generated due to in vitro digestion conditions [22,27], as these conformational changes (epimerization) in chlorophyll molecules are frequent with moderate heating [28], similar to the temperature used in the present study (37 °C) to simulate biological conditions. Additionally, the appearance of hydroxypheophytin a’ can occur through successive pheophytinization, allomerization and epimerization reactions from the native structure. However, no conclusion can be drawn about whether epimerization occurs preferentially in native chlorophylls or their Mg-free oxygenated derivatives [22]. Our results remain inconclusive, as hydroxypheophytin a’ appears, while hydroxychlorophyll a, chlorophyll a, and hydroxypheophytin a disappear after digestion.

A significant reduction of all compounds was observed in the micellar fraction after in vitro digestion. All-*E*-β-carotene (8.45 ± 0.15 µg·g^−1^) and all-*E*-equinenone (7.91 ± 0.10 µg·g^−1^) remained the major carotenoids, followed by all-*E*-zeaxanthin (7.10 ± 0.10 µg·g^−1^), all-*E*-neochrome (2.33 ± 0.5 µg·g^−1^), all-*E*-lutein (1.72 ± 0.03 µg·g^−1^) and 15*Z*-echinenone (0.74 ± 0.03 µg·g^−1^).

Two hypotheses (i and ii)can be considered to explain the higher micellar content of all-*E*-β-carotene in detriment to other xanthophylls: (i) The high content of unsaturated fatty acids in the biomass of *C. calcitrans* is one possible cause [29], as several studies indicate that the presence of more significant fractions of fatty acids than unsaturated ones promotes the micellarization of carotenes, while the presence of saturated fatty acids promotes the micellar incorporation of xanthophylls [30,31,32,33,34]; (ii) Although the cell wall was partially disrupted before digestion, some conjugations between xanthophylls and proteins may remain, making it difficult to transfer these carotenoids to micelles [16,20,35].

Although many factors need to be evaluated, a convergence in the literature towards greater micellar incorporation of xanthophylls is evident [36,37]. This trend is observed for total micellar carotenoids since the total xanthophylls (19.80 ± 0.30 µg·g^−1^) are approximately twice the total carotenes content (8.45 ± 0.15 µg·g^−1^).

Referring to the micellarized chlorophyll fraction, pheophytin a (482.10 ± 1.15 µg·g^−1^) remained the majority compound. According to the literature [22], chlorophylls are very susceptible and can change the digestive process, especially in acidic conditions. A first step in the metabolization of chlorophylls leads to the central perfusion of Mg in the structure, giving rise to pheophytins [38], which clarifies the predominance of pheophytin in the micellar fraction. Likewise, a recent study [27], associated this micellar predominance of pheophytin with the acidic conditions of the gastric phase (pH 2.5).

### 2.2. Relative Bioaccessibility

**Figure 1:**
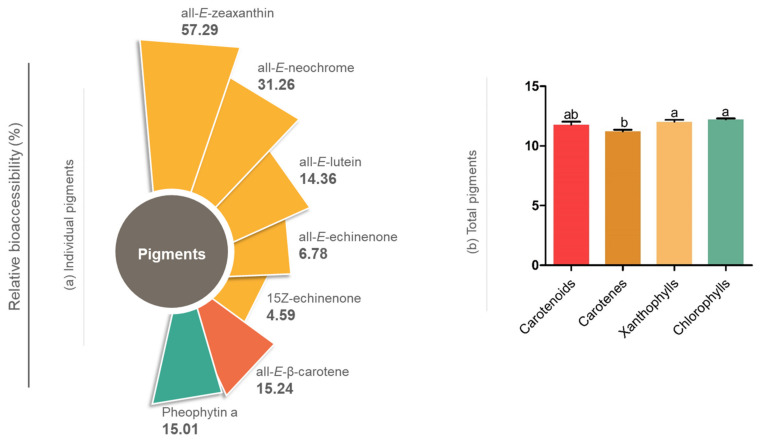
Relative bioaccessibility of individual (**a**) and total pigments (**b**) from *C. calcitrans*. Different letters in (**b**) indicate a significant difference using Tukey’s test (*p* < 0.05).

In terms of relative bioaccessibility (%) of *C. calcitrans* compounds (Figure 1a,b), there was a variation ranging from 4% to 58% (Figure 1a). Among individual compounds, the most bioaccessible carotenoid was all-*E*-zeaxanthin (57.29% ± 1.27), followed by all-*E*-neochrome (31.26% ± 0.42), all-*E*-β-carotene (15.24% ± 0.20), all-*E*-lutein (14.36% ± 0.33), all-*E*-echinenone (6.78% ± 0.01) and 15*Z*-echinenone (4.59% ± 0.16), while the only bioaccessible chlorophyll was pheophytin a (15.01% ± 0.20).

These bioaccessible compounds perform essential physiological and pharmacological activities which improve human health, well-being and nutritional status. These molecules are excellent antioxidants, reduce oxidative stress, benefit cardiovascular health. They also help prevent obesity, diabetes, some types of cancer, and neurological sequelae. In addition, some compounds such as β-carotene act precisely as vitamin A precursors, and zeaxanthin and lutein act as eye health regulators [39,40].

Comparatively, the relative bioaccessibility of all-*E*-zeaxanthin (57.29%) from *C. calcitrans* was superior to the findings for sonicated biomass from *Phaeadactylum tricornutum* (29%) [41], *Nannochloropsis* sp. (<15%) [20], *S. obliquus* (9%) [21], *Scenedesmus bijuga* (6%) [23], and a diet supplemented with *P. tricornutum* (17%) [41]. In addition, the bioaccessibility of all-*E*-β-carotene (15.24%) exceeded the values found in the sonicated biomass of *Chlorella vulgaris* (12%), *Chlamydomonas reinhardtii* (<10%) [19], *S. obliquus* (3%), *S. bijuga* (8%), and *Chlorella sorokiniana* (13%). Likewise, all-*E*-lutein (14.36%) surpassed the bioaccessibility found for sonicated biomass of *S. obliquus* (12%), *S. bijuga* (3%) and *C. sorokiniana* (6%) [21,23].

On the other hand, a study found bioaccessibility values of β-carotene of *P. tricornutum* up to 5 times higher than those established in this work [42]. Likewise, the lutein present in the diet supplemented with *C. vulgaris* was found to be approximately 2-fold higher [19]. The bioaccessibility of all-*E*-equinenone (6.78%) was similar to that found for sonicated biomass of *S. obliquus* (6%) [21]. However, we did not find comparative data for its 15*Z* isomer (4.59%) and all-*E*-neochrome (31.26%).

**Figure 2:**
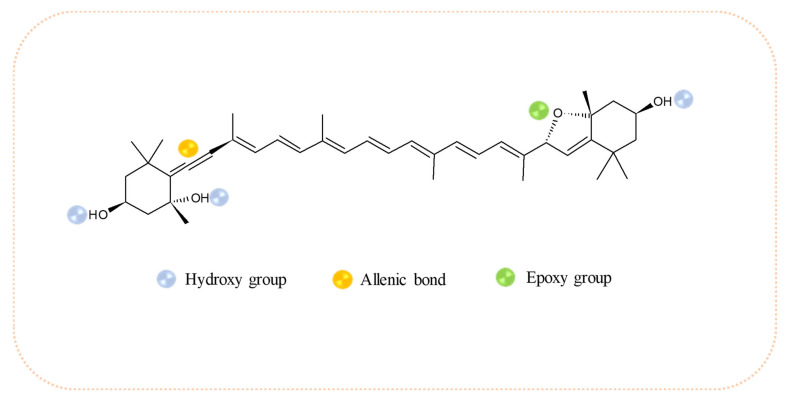
Chemical structure of the all-*E*-neochrome.

It is important to highlight that, as far as we know, this is the first time that the bioaccessibility of all-*E*-neochrome has been reported, a compound whose bioactive properties remain neglected, despite its remarkable structure (See Figure 2). In addition to some oxygenated functional groups (epoxy -O- and hydroxy -OH), neochrome has an unusual allenic bond (=C=), which has been implicated in increased deactivation of radical species when present in other isoprenoid structures [43].

The relative bioaccessibility of pheophytin a (15.01%) was higher than that reported for sonicated biomass of *S. obliquus* (~10%) [22]. Comparisons with literature data are extremely limited for the bioaccessibility of this microalgae compound group. Studies to date are scarce with only one recently published report [22].

Highlighting the totality of compounds, Figure 1b shows the total relative bioaccessibility for carotenoids (11.78% ± 0.25), carotenes (11.22% ± 0.23), xanthophylls (12.02% ± 0.26) and chlorophylls (12.22% ± 0.15). Xanthophylls and chlorophylls were slightly larger and differed significantly (*p* > 0.05) from the bioaccessible total carotenes. According to the literature, xanthophylls are generally more bioaccessible than carotenes due to their lower hydrophobicity [36,37].

When compared to different sources, the total bioaccessible chlorophyll of the sonicated biomass of *C. calcitrans* (12.22%) is four times greater than that of the sonicated biomass of *S. obliquus* (3%), for example, and is within the range determined for edible algae [22,44]. On the other hand, it is relatively low compared to experiments with isolated microalgae extracts (33%) or conventional sources (24–50%) [22,45].

As already demonstrated for different matrices, including microalgae, the transfer of carotenoids and chlorophylls to the micellar fraction can be influenced by numerous factors, especially the location in the matrix and the effect of constituents such as proteins, fatty acids, soluble fibers and minerals [23,46,47,48]. These factors may explain the differences observed in bioaccessibility studies of microalgae compounds to date, as the metabolic diversity of microalgae is immense, varies from species to species, and is still subject to modification according to the culture conditions, making it difficult to correlate all the variables involved.

Finally, when comparing the initial totality of compounds, both classes of pigments were reduced by more than 80% after mimicking digestion. This fact leads us to consider exploring alternatives to increase the micellarization efficiency and enhance its bioactive effects in vivo. The use of emulsions as a vehicle is an attractive option, mainly due to the increase in stability and incorporation of structures with non-polar characteristics in the micellar phase [3,21,49]. In addition, the inclusion of biomass in different food preparations should also be considered since integrated consumption is a future trend [4,50,51].

## 3. Material and Methods

### 3.1. Chemicals

The standards all-*E*-β-carotene, all-*E*-lutein, and chlorophyll a (with purities ranging from 95.0% to 99.9%), were purchased from Sigma-Aldrich (Darmstadt, Germany). All solvents for extraction and chromatography analysis were purchased from Merck (Darmstadt, Germany). The α-amylase (A3176), pepsin (P7000), pancreatin (P1750), lipase (L3126) and bile (B8631) were purchased from Sigma-Aldrich (St. Louis-MO, USA).

### 3.2. Microalgae Culture and Biomass Production

Axenic cultures of *Chaetoceros calcitrans* (CCMP1315) were used in the experiments. Stock cultures were propagated and maintained in BG-11 medium (Braun-Grunow medium) [52]. The incubation conditions included a temperature of 26 °C, a photon flux density of 15 μmol.m^−2^·s^−1^ and a photoperiod of 12 h.

The biomass productions were made in phototrophic conditions. The cultivations were performed in a bubble column photobioreactor under a batch regime fed on 2.0 L of BG-11 medium. The experimental conditions were as follows: initial cell concentration of 100 mg·L^−1^, isothermal reactor operating at a temperature of 26 °C, luminous intensity of 25 μmol.m^−2^.s^−1^, continuous aeration of 1 VVM (with air enriched with 3% CO_2_) and photoperiod of 24:0 h light/dark. The biomass was separated from the BG-11 medium by centrifugation (1500× *g*; 10 min; 10 °C), and the supernatant was discarded. The paste obtained after centrifugation was frozen at −18 °C for 24 h, and freeze-dried for 24 h at −50 °C above −175 μm Hg. The samples were stored under refrigeration until the analysis.

### 3.3. Sample Preparation

Before the in vitro digestion, aliquots of 100 mg of freeze-dried biomass were combined with 10 mL saline solution (NaCl 120 mol·L^−1^, CaCl_2_ 6 mmol·L^−1^, KCl 5 mmol·L^−1^) and were subjected to 15 min of an ultrasonic probe (Ultronic, Indaiatuba-SP, Brazil) to break the cell wall (an adaptation of Gille et al. [19]). The ultrasonic parameters were probe with 13 mm diameter, 400 W, 40 kHz, and an ice bath to control the temperature (0 ± 2 °C).

### 3.4. In Vitro Digestion

The samples were submitted to an in vitro simulated digestion model, according to the protocol adapted from INFOGEST [53] and modified by [27]. The oral phase was started with 6 mL of a solution of artificial saliva containing 106 U.mL^−1^ of α-amylase, followed by incubation at 37 °C, 10 min, 7.5× *g* in a shaking incubator (E-4200 model, Tecnal, Piracicaba, Brazil). Before starting the gastric phase, the pH was adjusted to 2.5 with HCl 1 mol L^−1^ followed by 2 mL of pepsin (50,000 U.mL^−1^ in HCl 100 mM). The total volume was adjusted to 40 mL, and the solution was incubated for 1 h, 37 °C, 7.5× *g* (E-4200 model, Tecnal, Piracicaba, Brazil). After this step, the pH was increased to 6.0 with 1M NaHCO_3_ and the intestinal phase started with a bile solution (3 mL; 40 mg.mL^−1^ in 100 mM NaHCO_3_), 4000 U.mL^−1^ of pancreatin and 1000 U.mL^−1^ of lipase. The pH was adjusted to 6.5 and the total volume to 50 mL, the incubation occurred for 2 h at 37 °C and 7.5× *g* (E-4200 model, Tecnal, Piracicaba, Brazil). After completion of the in vitro digestion, the solution was centrifuged at 8000× *g*, 60 min at 4 °C (Thermo, Langenselbold, Germany). The supernatant containing the mixed micelles was collected, covered with nitrogen gas, frozen at −40 °C and lyophilized for further extraction of pigments. The pigments bioaccessibility was calculated as the ratio between carotenoid content in the micellar fraction (supernatant) and original content in the *C. calcitrans* Equation (1).

(1) $$Bioaccessibility\;\left(\%\right)=\frac{Pigments\;\left(Supernatant\right)}{Pigments\;\left(original\;content\right)}\times 100$$

### 3.5. Pigments Extraction

The original content of *C. calcitrans* carotenoids and chlorophylls was extracted according to the literature [54]. The freeze-dried biomass (100 mg) was exhaustively extracted with ethyl acetate and methanol using a mortar and pestle followed by centrifugation (Thermo, Langenselbold, Germany) for 7 min at 1500× *g*. In addition, the carotenoids extract was saponified for 16 h with 10 g 100 mL^−1^ methanolic KOH at room temperature, and the alkali was removed by washing with distilled water. All extracts were concentrated in a rotary evaporator, placed in N_2_ atmosphere, and kept at −40 °C in the dark until analyzed.

The micellarized pigments were extracted according to an adapted protocol [55]. The lyophilized micellarized samples were exhaustively extracted by adding 15 mL of ethyl ether: petroleum ether (1:1) and subjected to 5 min ultrasonic cycles (see parameters in Section 3.3), centrifuged, and the supernatant was collected. The process was repeated until the supernatant became colorless. Then the carotenoids and chlorophyll extracts were rotary evaporated. The carotenoids extract underwent saponification as previously indicated. Both extracts were then in turn subjected to chromatographic analysis.

### 3.6. HPLC-PDA-MS/MS Pigments Analysis

The pigments were analyzed by high performance liquid chromatography HPLC (Shimadzu, Kyoto, Japan) equipped with binary pumps (model LC-20AD), online degasser, and automatic injector (Rheodyne, Rohnert Park-CA, USA). The chromatograph with photodiode array detection (PDA) (model SPD-M20A) was connected in series to an atmospheric pressure chemical ionization (APCI) source (Shimadzu America, Columbia, MD, USA), and a mass spectrometer Shimadzu 8040 triple quadrupole. The pigments separation was performed on a C30 YMC column (5 μm, 250 × 4.6 mm) (Waters, Wilmington-DE, USA). HPLC-PDA analysis was performed according to Rodrigues et al. [24]. Prior to HPLC-PDA analysis, the carotenoids extract was solubilized in methanol (MeOH): methyl tert-butyl ether (MTBE) (70:30) and filtered through Millipore membranes (0.22 μm). The mobile phases A (MeOH) and phase B (MTBE) used a linear gradient program as follows: from 0 to 30 min 5% B; from 30 to 40 min, 5 to 30% B; from 40 to 41 min, 30 to 50% B, from 41 to 50 min, 50 to 5% B. The flow rate was set at 0.9 mL.min^−1^, the injection volume was 20 μL, the column temperature was maintained at 29 °C, the UV-Vis spectra were acquired between 220 and 700 nm, and the chromatograms were processed at 450 nm.

The MS/MS analysis was conducted according to Giuffrida et al. [56] with adaptations: the APCI interface operated in positive (+) mode; detector voltage: 4.5 kV; interface temperature: 350 °C; DL temperature: 250 °C; heat block temperature: 200 °C; nebulizing gas flow (N_2_): 3.0 L.min^−1^; drying gas flow (N_2_): 5.0 L.min^−1^; collision-induced dissociation (CID) gas: 23 kPa (argon); event time: 0.5 s. To improve identification quality, MS/MS was used simultaneously in SIM (Select Ion Monitoring) and MRM (Multiple Reaction Monitoring) modes.

The identification was performed according to the following combined information: elution order on C30 HPLC column, co-chromatography with authentic standards, UV-Vis spectrum, and mass characteristics (protonated molecule ([M+H]^+^) and MS/MS fragments), compared with data available in the literature [21,25,26,57,58,59]. The pigments were individually quantified by HPLC-PDA using five-point calibration curves. The all-*E*-lutein, all-*E*-β-carotene end chlorophyll analytical curves were used to quantify the xanthophylls, carotenes and chlorophylls, respectively.

### 3.7. Statistical Analysis

The statistical analysis was performed using GraphPad Prism 5.0 software (GraphPad Software Inc., La Jolla-CA, USA). Differences between the two variables were detected by Student’s *t*-test (*p* < 0.05) and differences between more than two variables were assessed by a one-way ANOVA followed by Tukey’s test (*p* < 0.05).

## 4. Conclusions

This study investigated the bioaccessibility of carotenoids and chlorophylls from the diatom *C. calcitrans* for the first time. The relative bioaccessibility of sonicated biomass varied over a wide range (4–58%). The qualitative profile of bioaccessible carotenoids reflected the initial sample, with all-*E*-zeaxanthin (57.29%) being the major compound, followed by all-*E*-neochrome (reported for the first time in the micellar fraction). In contrast, pheophytin a (15.01%) was the only bioaccessible chlorophyll. Additionally, a chlorophyll derivative (hydroxypheophytin a’) was detected only in the micellar fraction. Considering all classes, xanthophylls (12.03%) and chlorophylls (12.22%) were significantly more bioaccessible than carotenes (11.28%). Although the considerable bioaccessibility of individual compounds is evidence for the bioactive potential of this source, the reduction of approximately 80% in the content of the compounds after in vitro digestion suggests that additional strategies to increase the micellarization efficiency are required in the future.

## Figures and Tables

**Table 1:** Chromatographic, UV–vis spectrum, mass characteristics of *C. calcitrans* pigments obtained by HPLC-PDA-MS/MS.

Pigments	t_R_^a^	UV-Vis Characteristics			Fragment Ions (Positive Mode) (*m*/*z*)	
		λ_máx_ (nm) ^b^	III/II (%) ^c^	AB/II (%) ^d^	[M+H]^+^	MS/MS
All-*E*-neochrome	5.23	399, 421, 448	94	0	601	583 [M+H−18]^+^, 491 [M+H−92−18]^+^
Hydroxychlorophyll a	10.17	430, 664	na ^e^	na	909	631 [M+H−278]^+^
All-*E*-lutein	12.64	418, 444, 473	50	0	569	551 [M+H−18]^+^, 533 [M+H−18−18]^+^, 495 [M+H−18−56]^+^, 477 [M+H−92]^+^, 459 [M+H−18−92]^+^
Chlorophyll a	15.11	432, 665	na	na	893	615 [M+H−278]^+^, 583 [M+H−278−31]^+^, 555 [M+H −278−59]^+^
Chlorophyll a’	16.73	431, 665	na	na	893	615 [M+H−278]^+^, 583 [M+H−278−31]^+^, 555 [M+H−278−59]^+^
All-*E*-zeaxanthin	17.51	421, 450, 477	25	0	569	551 [M+H−18]^+^, 533 [M+H−18−18]^+^,495, 477 [M+H−92]^+^, 459 [M+H−18−92]^+^
15*Z*-echinenone	19.09	335, 447	nc ^f^	20	551	533 [M+H−18]^+^, 427
Hydroxypheophytin a	24.10	409, 666	na	na	887	869 [M+H−18]^+^; 803 [M+H−63]^+^; 609 [M+H−278]^+^; 591 [M+H−278−18]^+^; 531 [M+H−278−18−60]^+^
All-*E*-echinenone	24.04	461	nc	nc	551	533 [M+H−18]^+^, 427
Hydroxypheophytin a’	27.91	399, 660	na	na	887	869 [M+H−18]^+^; 803 [M+H−63]^+^; 609 [M+H−278]^+^; 591 [M+H−278−18]^+^; 531 [M+H−278−18−60]^+^
All-*E-*β-carotene	32.14	424, 450, 476	28	0	537	481 [M+H−56]^+^, 444 [M−92]^+^, 413, 399, 355
Pheophytin a	32.87	408, 666	na	na	871	593 [M+H−278]^+^; 533 [M+H−278−60]^+^
9*Z*-β-carotene	34.22	353, 421, 443, 473	20	14	537	481 [M+H−56]^+^, 444 [M−92]^+^, 413, 399

^a^: Retention time (Linear gradient in methanol and methyl tert-butyl ether); ^b^: Spectral fine structure; ^c^: Ratio of the height of the longest wavelength absorption peak (III) and that of the middle absorption peak (II); ^d^: Ratio of the cis peak (AB) and the middle absorption peak (II); ^e^: Not applicable; ^f^: Not calculated.

**Table 2:** Pigment content of *C. calcitrans* before in vitro digestion (initial content), and the micellar fraction of carotenoids from ultrasonicated biomass after in vitro digestion. Different letters in the lines indicate a significant difference using Student’s *t*-test (*p* < 0.05).

Pigments	Initial Content (µg·g^−1^)	Micelar Fraction (µg·g^−1^)
All-*E*-neochrome	7.44 ± 0.05 ^a^	2.33 ± 0.5 ^b^
Hydroxychlorophyll a	221.99 ± 1.00	nd
All-*E*-lutein	12.01 ± 0.10 ^a^	1.72 ± 0.03 ^b^
Chlorophyll a	306.03 ± 1.04	nd
Chlorophyll a’	47.77 ± 0.69	nd
All-*E*-zeaxanthin	12.40 ± 0.10 ^a^	7.10 ± 0.10 ^b^
15*Z*-echinenone	16.06 ± 0.15 ^a^	0.74 ± 0.03 ^b^
Hydroxypheophytin a	110.85 ± 0.78	nd
All-*E*-echinenone	116.65 ± 1.52 ^a^	7.91 ± 0.10 ^b^
Hydroxypheophytin a’	Nd	20.89 ± 0.10
All-*E*-carotene	55.44 ± 0.25 ^a^	8.45 ± 0.15 ^b^
Pheophytin a	3257.52 ± 40.23 ^a^	482.10 ± 1.15 ^b^
9*Z*-β-carotene	19.83 ± 0.15	nd
Total carotenoids	239.88 ± 1.82 ^a^	28.24 ± 0.45 ^b^
Total carotenes	75.28 ± 0.40 ^a^	8.45 ± 0.15 ^b^
Total xanthophylls	164.61 ± 1.43 ^a^	19.80 ± 0.30 ^b^
Total chlorophylls	3944.16 ± 41.56 ^a^	502.15 ± 1.17 ^b^

nd: Not detected.

## Data Availability

Not applicable.

## Supplementary Materials

The following supporting information can be downloaded at: https://www.mdpi.com/article/10.3390/molecules27103305/s1, Figure S1: Representative chromatograms HPLC-PDA of Chaetoceros calcitrans carotenoids. Original content (control extract) before digestion (a). After in vitro digestion (b). See text for chromatographic conditions. The chromatogram was processed at 450 nm. 1. All-E-neochrome; 3. All-E-lutein; 6. All-E-zeaxanthin; 7. 15Z-echinenone; 9. All-E-echinenone; 11. All-E-β-carotene; 13. 9Z-β-carotene.; Figure S2: Representative chromatograms HPLC-PDA of Chaetoceros calcitrans chlorophylls. Original content (control extract) before digestion (a). After in vitro digestion (b). See text for chromatographic conditions. The chromatogram was processed at 660 nm. 2. Hydroxychlorophyll a; 4. Chlorophyll a; 5. Chlorophyll a’; 8. Hydroxypheophytin a; 10. Hydroxypheophytin a’; 12. Pheophytin a; Figure S3: PDA and MS-MS (MRM) spectra of some compounds identified from Chaetoceros calcitrans.

## Sample Availability

Samples of compounds are available from the authors.

## References

[1] Koyande A.K., Chew K.W., Rambabu K., Tao Y., Chu D.-T., Show P.-L. (2019). Microalgae: A potential alternative to health supplementation for humans. Food Sci. Hum. Wellness.

[2] Balasubramaniam V., Devi-Nair Gunasegavan R., Mustar S., Chelyn Lee J., Fairulnizal Mohd Noh M., Díaz-Marrero A.R., Javier Fernández Castro J. (2021). Isolation of Industrial Important Bioactive Compounds from Microalgae. Molecules.

[3] Ying Ying Tang D., Shiong Khoo K., Wayne Chew K., Tao Y., Ho S.-H., Loke Show P. (2020). Potential Utilization of Bioproducts from Microalgae for the Quality Enhancement of Natural Products. Bioresour. Technol..

[4] Tzachor A., Richards C.E., Holt L. (2021). Future foods for risk-resilient diets. Nat. Food.

[5] Jacob-Lopes E., Maroneze M.M., Deprá M.C., Sartori R.B., Dias R.R., Zepka L.Q. (2019). Bioactive food compounds from microalgae: An innovative framework on industrial biorefineries. Curr. Opin. Food Sci..

[6] Azizan A., Bustamam M.S.A., Maulidiani M., Shaari K., Ismail I.S., Nagao N., Abas F. (2018). Metabolite Profiling of the Microalgal Diatom *Chaetoceros Calcitrans* and Correlation with Antioxidant and Nitric Oxide Inhibitory Activities via 1H NMR-Based Metabolomics. Mar. Drugs.

[7] Foo S.C., Yusoff F.M., Ismail M., Basri M., Khong N.M.H., Chan K.W., Yau S.K. (2015). Efficient solvent extraction of antioxidant-rich extract from a tropical diatom, *Chaetoceros calcitrans* (Paulsen) Takano 1968. Asian Pac. J. Trop. Biomed..

[8] Mulders K.J., Lamers P.P., Martens D.E., Wijffels R.H. (2014). Phototrophic pigment production with microalgae: Biological constraints and opportunities. J. Phycol..

[9] Borowitzka M.A. (2018). Chapter 9—Microalgae in Medicine and Human Health: A Historical Perspective. Microalgae in Health and Disease Prevention.

[10] Rodriguez-Concepcion M., Avalos J., Bonet M.L., Boronat A., Gomez-Gomez L., Hornero-Mendez D., Limon M.C., Meléndez-Martínez A.J., Olmedilla-Alonso B., Palou A. (2018). A global perspective on carotenoids: Metabolism, biotechnology, and benefits for nutrition and health. Prog. Lipid Res..

[11] Zepka L.Q., Jacob-Lopes E., Roca M. (2019). Catabolism and bioactive properties of chlorophylls. Curr. Opin. Food Sci..

[12] Silva S.C., Ferreira I.C.F.R., Dias M.M., Filomena Barreiro M. (2020). Microalgae-Derived Pigments: A 10-Year Bibliometric Review and Industry and Market Trend Analysis. Molecules.

[13] do Nascimento T.C., Jacob-Lopes E., .Zepka L.Q. (2021). Microalgas e saúde: Uma breve revisão/Microalgae and health: A short-review. Braz. J. Dev..

[14] Kusmayadi A., Leong Y.K., Yen H.-W., Huang C.-Y., Chang J.-S. (2021). Microalgae as sustainable food and feed sources for animals and humans—Biotechnological and environmental aspects. Chemosphere.

[15] Mehariya S., Goswami R.K., Karthikeysan O.P., Verma P. (2021). Microalgae for high-value products: A way towards green nutraceutical and pharmaceutical compounds. Chemosphere.

[16] Kopec R.E., Failla M.L. (2018). Recent advances in the bioaccessibility and bioavailability of carotenoids and effects of other dietary lipophiles. J. Food Compos. Anal..

[17] Fernandes A.S., Jacob-Lopes E., Zepka L.Q. (2021). An overview on microalgae carotenoids and chlorophylls: Focus in the bioaccessibility/Uma visão geral dos carotenoides e clorofilas microalgais: Foco na bioacessibilidade. Braz. J. Dev..

[18] Carbonell-Capella J.M., Buniowska M., Barba F.J., Esteve M.J., Frígola A. (2014). Analytical methods for determining bioavailability and bioaccessibility of bioactive compounds from fruits and vegetables: A review. Compr. Rev. Food Sci. Food Saf..

[19] Gille A., Trautmann A., Posten C., Briviba K. (2016). Bioaccessibility of carotenoids from *Chlorella vulgaris* and *Chlamydomonas reinhardtii*. Int. J. Food Sci. Nutr..

[20] Bernaerts T.M.M., Verstreken H., Dejonghe C., Gheysen L., Foubert I., Grauwet T., Van Loey A.M. (2020). Cell disruption of *Nannochloropsis* sp. improves in vitro bioaccessibility of carotenoids and ω3-LC-PUFA. J. Funct. Foods.

[21] do Nascimento T.C., Pinheiro P.N., Fernandes A.S., Murador D.C., Neves B.V., de Menezes C.R., de Rosso V.V., Jacob-Lopes E., Zepka L.Q. (2021). Bioaccessibility and intestinal uptake of carotenoids from microalgae Scenedesmus obliquus. LWT.

[22] Fernandes A.S., Nascimento T.C., Pinheiro P.N., de Rosso V.V., de Menezes C.R., Jacob-Lopes E., Zepka L.Q. (2021). Insights on the intestinal absorption of chlorophyll series from microalgae. Food Res. Int..

[23] Fernandes A.S., Nascimento T.C., Pinheiro P.N., Vendruscolo R.G., Wagner R., de Rosso V.V., Jacob-Lopes E., Zepka L.Q. (2021). Bioaccessibility of microalgae-based carotenoids and their association with the lipid matrix. Food Res. Int..

[24] Rodrigues D.B., Menezes C.R., Mercadante A.Z., Jacob-lopes E., Zepka L.Q. (2015). Bioactive pigments from microalgae *Phormidium autumnale*. Food Res. Int..

[25] Fernandes A.S., Nogara G.P., Menezes C.R., Cichoski A.J., Mercadante A.Z., Jacob-Lopes E., Zepka L.Q. (2017). Identification of chlorophyll molecules with peroxyl radical scavenger capacity in microalgae *Phormidium autumnale* using ultrasound-assisted extraction. Food Res. Int..

[26] Fernandes A.S., Petry F.C., Mercadante A.Z., Jacob-Lopes E., Zepka L.Q. (2020). HPLC-PDA-MS/MS as a strategy to characterize and quantify natural pigments from microalgae. Curr. Res. Food Sci..

[27] Murador D.C., Mesquita L.M.D.S., Neves B.V., Braga A.R.C., Martins P.L.G., Zepka L.Q., Rosso V.V. (2020). De Bioaccessibility and Cellular Uptake by Caco-2 Cells of Carotenoids and Chlorophylls from Orange Peels: A Comparison Between Conventional and Ionic Liquid Mediated Extractions. Food Chem..

[28] Gauthier-Jaques A., Bortlik K., Hau J., Fay L.B. (2001). Improved Method to Track Chlorophyll Degradation. J. Agric. Food Chem..

[29] Servel M.O., Claire C., Derrien A., Coiffard L., De Roeck-Holtzhauer Y. (1994). Fatty acid composition of some marine microalgae. Phytochemistry.

[30] Failla M.L., Chitchumronchokchai C., Ferruzzi M.G., Goltz S.R., Campbell W.W. (2014). Unsaturated fatty acids promote bioaccessibility and basolateral secretion of carotenoids and α-tocopherol by Caco-2 cells. Food Funct..

[31] Yuan X., Liu X., McClements D.J., Cao Y., Xiao H. (2018). Enhancement of phytochemical bioaccessibility from plant-based foods using excipient emulsions: Impact of lipid type on carotenoid solubilization from spinach. Food Funct..

[32] Nagao A., Kotake-Nara E., Hase M. (2013). Effects of fats and oils on the bioaccessibility of carotenoids and vitamin E in vegetables. Biosci. Biotechnol. Biochem..

[33] Zhang R., Zhang Z., Zou L., Xiao H., Zhang G., Decker E.A., McClements D.J. (2015). Enhancing Nutraceutical Bioavailability from Raw and Cooked Vegetables Using Excipient Emulsions: Influence of Lipid Type on Carotenoid Bioaccessibility from Carrots. J. Agric. Food Chem..

[34] Han J.R., Gu L.P., Zhang R.J., Shang W.H., Yan J.N., McClements D.J., Wu H.T., Zhu B.W., Xiao H. (2019). Bioaccessibility and cellular uptake of β-carotene in emulsion-based delivery systems using scallop (*Patinopecten yessoensis*) gonad protein isolates: Effects of carrier oil. Food Funct..

[35] Chitchumroonchokchai C., Failla M.L. (2017). Bioaccessibility and intestinal cell uptake of astaxanthin from salmon and commercial supplements. Food Res. Int..

[36] Berni P., Campoli S.S., Negri T.C., de Toledo N.M.V., Canniatti-Brazaca S.G. (2019). Non-conventional Tropical Fruits: Characterization, Antioxidant Potential and Carotenoid Bioaccessibility. Plant Foods Hum. Nutr..

[37] Petry F.C., Mercadante A.Z. (2017). Impact of in vitro digestion phases on the stability and bioaccessibility of carotenoids and their esters in mandarin pulps. Food Funct..

[38] Huo T., Ferruzzi M.G., Schwartz S.J., Failla M.L. (2007). Impact of fatty acyl composition and quantity of triglycerides on bioaccessibility of dietary carotenoids. J. Agric. Food Chem..

[39] Eggersdorfer M., Wyss A. (2018). Carotenoids in human nutrition and health. Arch. Biochem. Biophys..

[40] Arunkumar R., Gorusupudi A., Bernstein P.S. (2020). The macular carotenoids: A biochemical overview. Biochim. Biophys. Acta—Mol. Cell Biol. Lipids.

[41] Gille A., Neumann U., Louis S., Bischoff S.C., Briviba K. (2018). Microalgae as a potential source of carotenoids: Comparative results of an in vitro digestion method and a feeding experiment with C57BL/6J mice. J. Funct. Foods.

[42] Gille A., Hollenbach R., Trautmann A., Posten C., Briviba K. (2019). Effect of sonication on bioaccessibility and cellular uptake of carotenoids from preparations of photoautotrophic *Phaeodactylum tricornutum*. Food Res. Int..

[43] Sachindra N.M., Sato E., Maeda H., Hosokawa M., Niwano Y., Kohno M., Miyashita K. (2007). Radical scavenging and singlet oxygen quenching activity of marine carotenoid fucoxanthin and its metabolites. J. Agric. Food Chem..

[44] Chen K., Roca M. (2018). In vitro digestion of chlorophyll pigments from edible seaweeds. J. Funct. Foods.

[45] Hayes M., Pottorff M., Kay C., Van Deynze A., Osorio-Marin J., Lila M.A., Iorrizo M., Ferruzzi M.G. (2020). In Vitro Bioaccessibility of Carotenoids and Chlorophylls in a Diverse Collection of Spinach Accessions and Commercial Cultivars. J. Agric. Food Chem..

[46] O’Connell O.F., Ryan L., O’Brien N.M. (2007). Xanthophyll carotenoids are more bioaccessible from fruits than dark green vegetables. Nutr. Res..

[47] Corte-Real J., Bohn T. (2018). Interaction of divalent minerals with liposoluble nutrients and phytochemicals during digestion and influences on their bioavailability—A review. Food Chem..

[48] Granado-Lorencio F., Herrero-Barbudo C., Acién-Fernández G., Molina-Grima E., Fernández-Sevilla J.M., Pérez-Sacristán B., Blanco-Navarro I. (2009). In vitro bioaccesibility of lutein and zeaxanthin from the microalgae *Scenedesmus almeriensis*. Food Chem..

[49] Xavier A.A.O., Mercadante A.Z. (2019). The bioaccessibility of carotenoids impacts the design of functional foods. Curr. Opin. Food Sci..

[50] Lafarga T., Rodríguez-Bermúdez R., Morillas-España A., Villaró S., García-Vaquero M., Morán L., Sánchez-Zurano A., González-López C.V., Acién-Fernández F.G. (2021). Consumer knowledge and attitudes towards microalgae as food: The case of Spain. Algal Res..

[51] Lafarga T. (2019). Effect of microalgal biomass incorporation into foods: Nutritional and sensorial attributes of the end products. Algal Res..

[52] Rippka R., Deruelles J., Waterbury J.B., Herdman M., Stanier R.Y. (1979). Generic assignments, strain histories and properties of pure cultures of cyanobacteria. J. Gen. Microbiol..

[53] Minekus M., Alminger M., Alvito P., Ballance S., Bohn T., Bourlieu C., Carrière F., Boutrou R., Corredig M., Dupont D. (2014). A standardised static in vitro digestion method suitable for food—An international consensus. Food Funct..

[54] Mandelli F., Miranda V.S., Rodrigues E., Mercadante A.Z. (2012). Identification of carotenoids with high antioxidant capacity produced by extremophile microorganisms. World J. Microbiol. Biotechnol..

[55] Ordóñez-Santos L.E., Pinzón-Zarate L.X., González-Salcedo L.O. (2015). Optimization of ultrasonic-assisted extraction of total carotenoids from peach palm fruit (*Bactris gasipaes*) by-products with sunflower oil using response surface methodology. Ultrason. Sonochem..

[56] Giuffrida D., Zoccali M., Giofrè S.V., Dugo P., Mondello L. (2017). Apocarotenoids determination in *Capsicum chinense* Jacq. cv. Habanero, by supercritical fluid chromatography-triple-quadrupole/mass spectrometry. Food Chem..

[57] De Rosso V.V., Mercadante A.Z. (2007). HPLC-PDA-MS/MS of anthocyanins and carotenoids from dovyalis and tamarillo fruits. J. Agric. Food Chem..

[58] Rodrigues D.B., Flores É.M.M., Barin J.S., Mercadante A.Z., Jacob-Lopes E., Zepka L.Q. (2014). Production of carotenoids from microalgae cultivated using agroindustrial wastes. Food Res. Int..

[59] Erdoğan A., Çağır A., Dalay M.C., Eroğlu A.E. (2015). Composition of Carotenoids in Scenedesmus protuberans: Application of Chromatographic and Spectroscopic Methods. Food Anal. Methods.

