# Supplementary material for: Insights on the Bioaccessibility of Natural Pigments from Diatom Chaetoceros calcitrans
Source: Molecules. 2022 May 21;27(10):3305. doi: 10.3390/molecules27103305 (PMC9147772; doi:10.3390/molecules27103305)

Figure S1: Representative chromatograms HPLC-PDA of *Chaetoceros calcitrans* carotenoids. Original content (control extract) before digestion (a). After *in vitro* digestion (b). See text for chromatographic conditions. The chromatogram was processed at 450 nm. 1. All-*E*-neochrome; 3. All-*E*-lutein; 6. All-*E*-zeaxanthin; 7. 15-*Z*-echinenone; 9. All-*E*-echinenone; 11. All-*E*-β-carotene; 13. 9-*Z*-β-carotene.

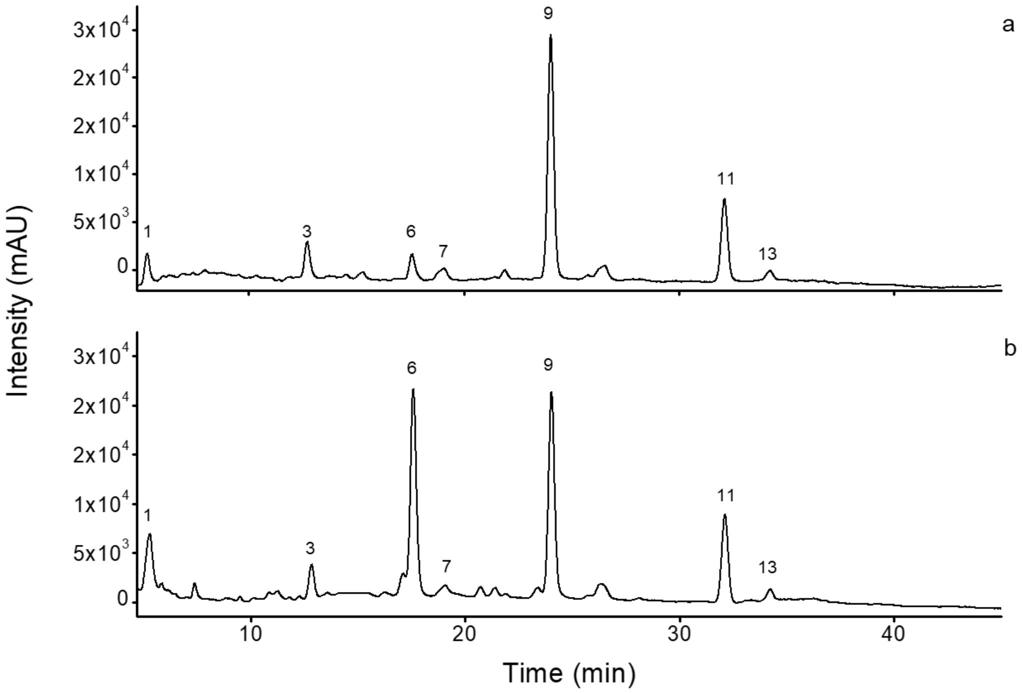

Figure S2: Representative chromatograms HPLC-PDA of *Chaetoceros calcitrans* chlorophylls. Original content (control extract) before digestion (a). After *in vitro* digestion (b). See text for chromatographic conditions. The chromatogram was processed at 660 nm. 2. Hydroxychlorophyll a; 4. Chlorophyll a; 5. Chlorophyll a′; 8. Hydroxypheophytin a; 10. Hydroxypheophytin a′; 12. Pheophytin a.

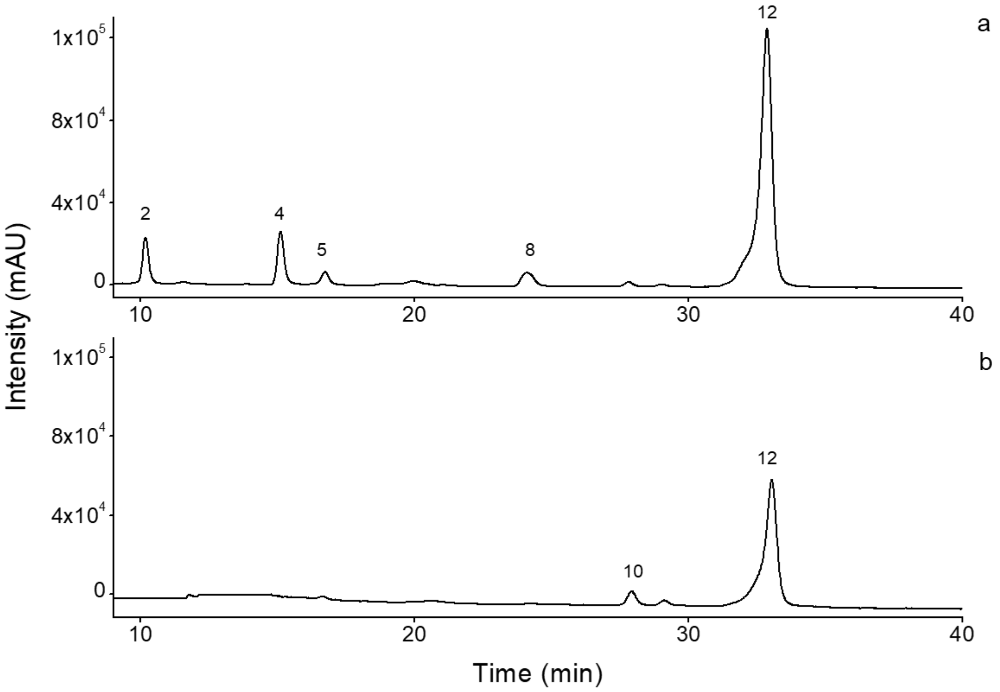

Figure S3: PDA and MS-MS (MRM) spectra of some compounds identified from *Chaetoceros calcitrans*.

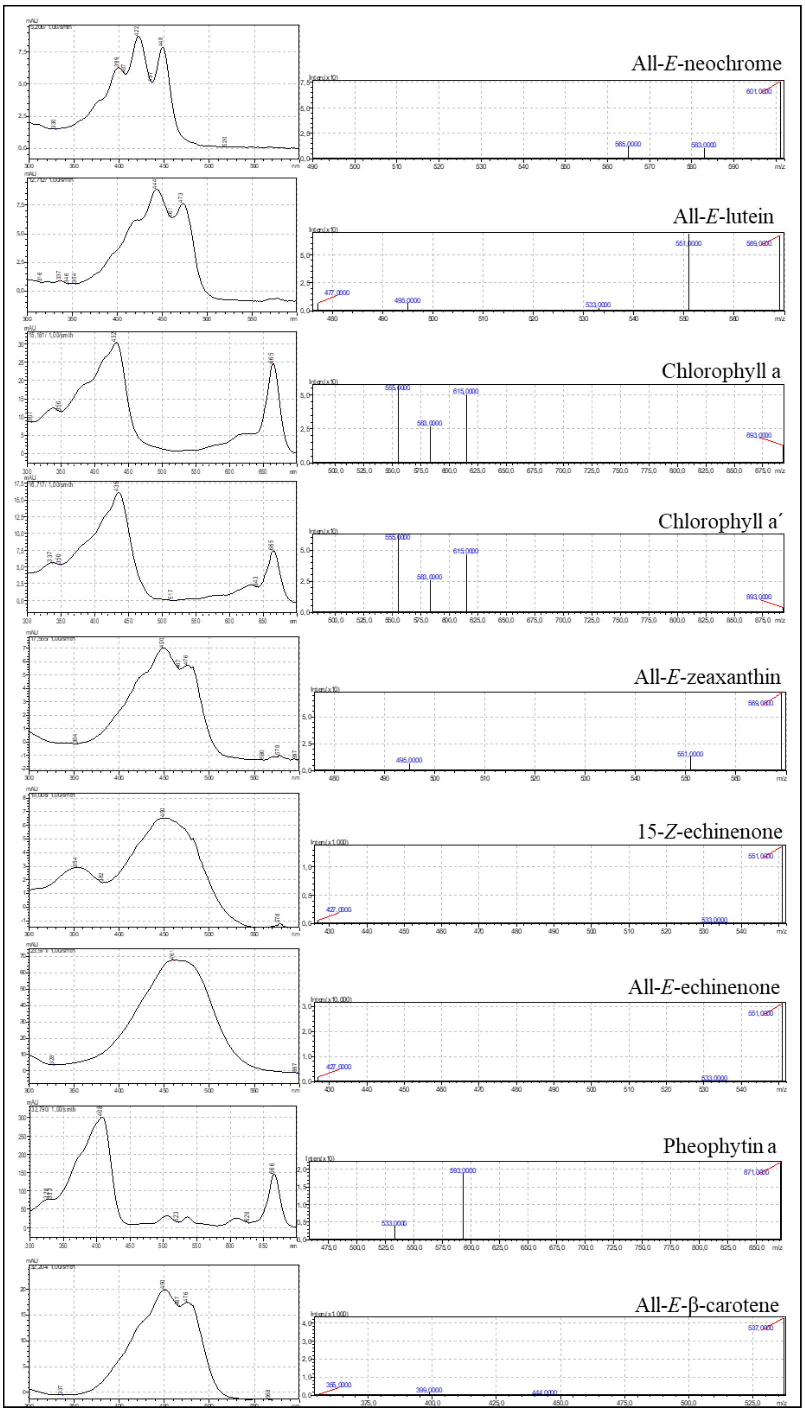

Supplement: Supplementary file 1 [file molecules-27-03305-s001.zip › molecules-1613154-supplementary.pdf]
